# *Fusobacterium nucleatum* promotes metastasis of breast cancer via the miR-21-3p/FOXO3 axis

**DOI:** 10.3389/fonc.2025.1530269

**Published:** 2025-06-16

**Authors:** Yiping Huang, Zhongzhong Guo, Zhaoyou Zeng, Chenyu Shang, Yingfeng Zhang, Ziwei Ran, Guoqing Luo, Sandi Shen, Yaqin Liu, Peng Zhou, Peng Ma, Yanxia Zhang, Haibiao Lin, Yang Lu, Dongdong Liu

**Affiliations:** ^1^ School of Public Health, Dali University, Dali, Yunnan, China; ^2^ Division of Gastroenterology, Institute of Digestive Disease, The Affiliated Qingyuan Hospital, Qingyuan People’s Hospital, Guangzhou Medical University, Qingyuan, Guangdong, China; ^3^ The Affiliated Qingyuan Hospital, Qingyuan People’s Hospital, Guangzhou Medical University, Qingyuan, Guangdong, China; ^4^ Department of Laboratory Medicine, The Second Affiliated Hospital of Guangzhou University of Chinese Medicine, Guangzhou, China; ^5^ State Key Laboratory of Dampness Syndrome of Chinese Medicine, The Second Affiliated Hospital of Guangzhou University of Chinese Medicine, Guangzhou, China

**Keywords:** breast cancer, *Fusobacterium nucleatum*, miR-21-3p, FOXO3, metastasis

## Abstract

**Purpose:**

This study aims to investigate the impact of Fusobacterium nucleatum (F. nucleatum) infection on distant metastasis in breast cancer and its underlying mechanisms.

**Materials:**

Clinical breast cancer samples were collected, and F. nucleatum was identified through bacterial isolation and culture. In vitro and in vivo infection models were established using the isolated bacteria. The impact of F. nucleatum infection on breast cancer cell proliferation and migration was evaluated. High-throughput sequencing and bioinformatics analyses were performed to identify microRNAs exhibiting significant expression changes following F. nucleatum infection. Gene knockdown of miR-21-3p was utilized to assess its role in F. nucleatum-mediated epithelial-mesenchymal transition and cell migration. Online databases predicted the downstream targets of miR-21-3p, which were subsequently validated in cell models. Additionally, the effect of silencing FOXO3 on F. nucleatum-induced cell migration was examined.

**Results:**

Both in vitro and in vivo experiments showed that F. nucleatum infection did not affect cell proliferation but significantly enhanced EMT and migration. miR-21-3p was significantly upregulated after infection, and silencing it reduced F. nucleatum-induced migration. FOXO3 was identified as a downstream target of miR-21-3p, and silencing FOXO3 further enhanced cell migration.

**Conclusion:**

F. nucleatum promotes breast cancer cell migration through the miR-21-3p/FOXO3 pathway. This study highlights the role of F. nucleatum in breast cancer metastasis and suggests potential therapeutic targets for intervention.

## Introduction

Breast cancer (BC) is among the most prevalent malignant tumors in women. In 2020, approximately 19.3 million new cancer cases were reported worldwide, with about 2.3 million of these being new diagnoses of female BC, accounting for 11.7% of all cancer cases. This incidence has surpassed that of lung cancer, positioning BC as the most prevalent type of cancer globally (1). In China, the number of newly diagnosed BC cases rose from 300,000 in 2015 to 420,000 in 2020 (2), accompanied by a steady increase in mortality rates. Distant metastasis remains the leading cause of this rising mortality. While the five-year survival rate for localized BC exceeds 99%, survival rates decline sharply following metastasis, especially in cases of distant spread, where the survival rate drops to merely 26% (3). Moreover, distant metastasis is a key factor contributing to treatment failure in BC patients. Therefore, thorough and systematic research into the molecular mechanisms underlying BC metastasis is essential for identifying more effective molecular targets and developing novel therapeutic strategies.

Tumor metastasis is closely associated with the acquisition of mesenchymal characteristics by epithelial cancer cells, a process referred to as EMT. In most cancers, excluding cancer stem cells, the majority of cancer cells lack the ability to colonize distant sites and form new lesions (4). In contrast, cancer cells that undergo EMT can exhibit cancer stem cell-like properties (5, 6) Therefore, EMT facilitates the migration of epithelial cancer cells to distant sites, subsequently initiating secondary tumor growth (4). Moreover, cancer cells undergoing EMT display increased resistance to treatment (7, 8). Thus, the identification of factors that influence EMT and their underlying mechanisms has emerged as a focal point in the study of tumor metastasis.

Initially, tumor tissue was hypothesized to exist in a sterile state. However, advancements in sequencing technology have progressively shifted attention toward intratumoral bacteria. In 2020, Ravid Straussman’s team was the first to map the “tumor microbiome” across seven types of tumors, analyzing over 1,000 samples. Their findings revealed the presence of diverse bacteria in bone cancer, brain cancer, ovarian cancer, BC, skin cancer, pancreatic cancer, and lung cancer, with positive detection rates reaching up to 60% (9). This discovery has sparked significant interest in exploring the types and roles of bacteria within tumors, leading to a series of new scientific insights. In 2021, Maria Rescigno’s research team described that Escherichia coli could alter the liver microenvironment by releasing virulence factors that disrupt the vascular barrier and attract metastasis-associated cells, thus creating favorable conditions for liver metastasis in colorectal cancer (10). Subsequently, a 2022 study led by Cai Shang, published in Cell, further indicated that intratumoral bacteria can assist BC cells in resisting fluid shear stress within blood vessels, thereby promoting their migration to the lungs (11). These studies highlight the crucial role of intratumoral bacteria in tumor metastasis. Nonetheless, research on intratumoral bacteria remains in its infancy, and the functions and mechanisms of these bacteria remain to be fully elucidated.

*Fusobacterium nucleatum* (*F. nucleatum*) is a common opportunistic pathogen that can colonize the human body via the gastrointestinal tract. Earlier studies have established that *F. nucleatum* is ubiquitous in various tumors and can influence tumorigenesis through multiple mechanisms. Its presence has been detected in the tissues of colorectal cancer (12), esophageal cancer (13), gastric cancer (14), cervical cancer (15), and BC (16). In colorectal cancer cells, *F. nucleatum* infection up-regulates the expression of miR-21 through the TLR4/MYD88/NF-κB signaling pathway, thereby facilitating cancer proliferation and metastasis (17). esearch conducted by Professor Zhang Ge’s team also demonstrated that *F. nucleatum* infection induces the synthesis of exosomes enriched with miR-1246/92b-3p/27a-3p and CXCL16/RhoA/IL-8, promoting colorectal cancer metastasis through various target molecules (18). Additionally, virulence factors of *F. nucleatum*, such as Adhesin A, Fusobacterium autotransporter protein 2, and Fusobacterium outer membrane protein A, enable the bacterium to adhere to and invade endothelial and epithelial cells (19–21), thus participating in carcinogenic pathways. In BC, studies have evinced that the autotransporter protein secreted by *F. nucleatum* can inhibit the accumulation of tumor-infiltrating T cells, thereby driving tumor growth and metastasis (22). In summary, while *F. nucleatum* plays a significant regulatory role in tumor metastasis, the specific mechanisms by which it influences BC metastasis remain elusive and require further investigation.

MicroRNAs (miRNAs) are a family of endogenous non-coding RNAs, approximately 21 nucleotides in length, that regulate various cellular activities, including development, differentiation, apoptosis, and cell cycle progression (23). Recent studies have validated that they play a decisive role in mediating distant metastasis in BC. For example, BC cells can secrete miR-105, which targets tight junction protein 1, thereby increasing vascular permeability and facilitating the migration of tumor cells through the bloodstream (24). Additionally, the high expression of miR-21 in BC tissues significantly promotes the colonization and metastasis of BC cells to the bone (25). Thus, miRNAs are essential regulatory factors in BC metastasis and may serve as potential biomarkers. However, their mediating effects on intratumoral bacteria in BC metastasis remain unknown.

Notably, *F. nucleatum* promotes distant metastasis in BC. However, whether this metastasis is mediated through the regulation of miRNAs remains unclear. Therefore, in this study, *F. nucleatum* was isolated from clinical samples, following which an *in vitro* infection model was constructed. Functional studies revealed that *F. nucleatum* significantly enhanced EMT and promoted the migration of breast cancer cells, consistent with the findings of prior investigations. Subsequently, miRNAs related to BC metastasis were identified via sequencing, and their expression levels in the infection model were validated. Notably, miR-21-3p was markedly upregulated in breast cancer cells following *F. nucleatum* infection. Conversely, miR-21-3p knockdown significantly inhibited *F. nucleatum*-induced EMT and migration. Further exploration using databases to identify downstream target genes of miR-21-3p, along with validation in cell models, identified Forkhead box class O3 (FOXO3) as a downstream target gene involved in *F. nucleatum*-mediated enhancement of EMT and migration in breast cancer cells. Collectively, these findings elucidate the role of the miR-21-3p/FOXO3 signaling axis in *F. nucleatum*-promoted BC metastasis, providing experimental evidence for further elucidating the pathogenic mechanisms of *F. nucleatum*.

## Materials and methods

### Sample collection and preparation

Fresh tissue samples from both tumor and adjacent normal tissues used in this study were obtained from The Medical Ethics Committee of the Affiliated Qinyuan Hospital of Guangzhou Medical University (Qinyuan People’s Hospital). The use of tumor samples was approved by the hospital’s Institutional Review Board (IRB-2024-068), and informed consent was obtained from all patients. The tissues were collected in a sterile operating room at Qinyuan People’s Hospital and immediately placed in 50 ml conical tubes containing sterile DMEM culture medium. Subsequent procedures were carried out using high-pressure sterilized dissection tools within a laminar flow hood to minimize contamination.

### Cell lines and bacteria

*F. nucleatum* strains were isolated and maintained by our research group. The cell lines MD Anderson-Metastatic Breast-231 (MDA-MB-231), Michigan Cancer Foundation-7 (MCF-7), and human umbilical vein endothelial cells (HUVECs) were also previously preserved by our research group.

Isolation, Cultivation, and Identification of Intratumoral Bacteria

Fresh clinical tissue samples were placed into 6 cm cell culture dishes containing DMEM culture medium. The tissues were minced using high-pressure sterilized surgical scissors and then transferred to 1.5 mL centrifuge tubes, following which they were thoroughly homogenized using a tissue disruptor. Next, a 100 μL aliquot of the tissue homogenate was inoculated onto blood agar plates, Sabouraud agar plates, and chocolate agar plates using sterile swabs to ensure even distribution. Additionally, 100 μL of the homogenate was added to the nutrient broth. Each of the four types of media was prepared in duplicate and incubated under both aerobic and anaerobic conditions at 37°C for up to 15 days. Visible colonies were selected for identification using mass spectrometry, and the strains were preserved at -80°C.

### *F. nucleatum* infection of BC cells

Frozen *F. nucleatum* stocks were thawed and cultured on blood agar under anaerobic conditions at 37°C for 48 hours. Afterward, single colonies were suspended in pure DMEM to prepare a bacterial suspension, which was subsequently counted and used for infection. The BC cell lines, namely MDA-MB-231 and MCF-7, were digested into single-cell suspensions the day before infection, seeded in cell culture plates, and grown to 70-80% confluence. *F. nucleatum* was added at a multiplicity of infection (MOI) of 50:1, gently mixed, and incubated for 48 hours in a cell culture incubator.

### CCK-8 cell proliferation assay

Approximately 1 × 10^4^ BC cells were seeded in 96-well plates and allowed to adhere. The cells were then infected with *F. nucleatum* at an MOI of 50:1. At 0, 24, 48, and 72 hours of culture, the old media were replaced with fresh DMEM containing 10% FBS after washing with PBS. CCK-8 reagent (DOJINDO) was added, and the cells were incubated for approximately 1 hour. Absorbance was measured at 450 nm using a microplate reader to generate growth curves.

### Transwell migration assay

Upon the BC cell density reaching approximately 60% in 6-well plates, the cells were infected with *F. nucleatum* at an MOI of 50:1 for 48 hours. After infection, the cells were detached, centrifuged, resuspended in serum-free DMEM, and adjusted to a concentration of 2.5 × 10^5^ cells/mL. Thereafter, 200 μL of the cell suspension was added to the upper chamber of the Transwell inserts, while 800 μL of complete DMEM supplemented with 20% FBS was added to the lower chamber. The plates were incubated at 37°C in 5% CO2 for 24–36 hours, then fixed with 4% paraformaldehyde and stained with crystal violet. Cells on the upper surface of the membrane were gently wiped off, and cell migration was observed and photographed under a microscope.

### Wound healing assay

BC cells were seeded in 6-well plates and grown to confluence. Horizontal lines were marked on the back of the plates at 0.5 cm intervals, with three lines per well. After infection with *F. nucleatum* at an MOI of 50:1 for 48 hours, scratches were made perpendicular to the horizontal lines using a 200 µL pipette tip. Floating cells were discarded using PBS, and the medium was replaced with 2 mL of serum-free DMEM. The plates were photographed under an inverted microscope at 0 hours and after 24 hours of incubation. Scratch areas were analyzed using Image J software.

### Real-time quantitative PCR

Total RNA from tissues or cells was extracted using an RNA extraction kit, followed by reverse transcription into cDNA using Evo M-MLV reverse transcription premix (Aker Bio). The expression levels of mRNA (Aker Bio) and miRNAs (Ribobio) were then analyzed. Specific primers used for the analysis are listed in the **Supplementary Materials** (**Supplementary Table 1**).

### Western blotting

When cell confluence exceeded 90%, the culture medium was removed, and the cells were washed twice with PBS. RIPA lysis buffer with a 1% protease inhibitor mixture was introduced (200 µL per well in a 6-well plate). After complete lysis, the cells were scraped using a cell scraper and transferred to 1.5 mL EP tubes. The lysates were sonicated on ice and centrifuged at 12,000 rpm for 10 minutes at 4°C, and the supernatants were collected. Protein concentration was determined using a BCA assay kit (Beyotime). Western blot analysis was performed using antibodies against GAPDH (CST), β-actin (CST), FOXO3 (CST), E-cadherin (CST) and Vimentin (CST), followed by detection using a chemiluminescence system. The results were photographed and saved.

### Cell transfection

When BC cells in the 12-well plate reached a confluency of 60%-80%, 100 μL of the serum-free medium was added to a sterile, enzyme-free 1.5 mL EP tube. This was followed by the addition of 2.5 μL of miR-21-3p inhibitor/inhibitor NC (Ribiotech) or FOXO3 siRNA/siRNA NC (Ribiotech), with the corresponding sequences provided in the **Supplementary Materials** (**Supplementary Table 2**). The mixture was gently mixed. Meanwhile, the Simple-Pect Transfection Reagent was allowed to equilibrate to room temperature. Subsequently, 4.5 μL of the transfection reagent was added to the RNA mixture, gently mixed, and allowed to stand at room temperature for 15–20 minutes. During this incubation period, the cell culture medium in the 12-well plate was discarded and replaced with 400 μL of fresh medium containing 10% serum. After the incubation period, the RNA-transfection reagent complex was immediately added to the 12-well plate, ensuring even cell coverage. The plate was then incubated at 37°C with 5% CO2 for 6 hours, after which the complex was removed, and 1 mL of culture medium was added. The cells were further incubated at 37°C with 5% CO2. RNA or protein was extracted for subsequent experiments 48 hours post-transfection.

### Bioinformatics analysis

The Cancer Genome Atlas (TCGA) (26) BRCA expression profile data were downloaded from the UCSC XENA database (https://xena.ucsc.edu/) (27). The CancerMIRNome database (http://bioinfo.jialab-ucr.org/CancerMIRNome) (28) was utilized to examine the expression levels of miR-21-3p in BC and to evaluate its diagnostic potential. The miRDB online database (https://mirdb.org/) (29) was utilized to predict the binding sites between miR-21-3p and the FOXO3 gene. The Kaplan-Meier Plotter database (https://kmplot.com/analysis/) (30) was used to assess the impact of miR-21-3p expression on overall survival in BC patients.

### Statistical analysis

All experiments were conducted in triplicate, and data analysis was carried out using GraphPad Prism 8 software. Independent sample t-tests were applied to compare data between two experimental groups, while one-way analysis of variance (ANOVA) was used for comparisons among multiple groups. Western blot grayscale values were analyzed through Image J software, and graphs were generated using GraphPad Prism 8. Statistical significance was denoted as follows: * for *p* < 0.05, ** for *p* < 0.01, and *** for *p* < 0.001.

## Results

### *F. nucleatum* is present in BC tissues and promote the migration of BC cells

**Figure 1:**
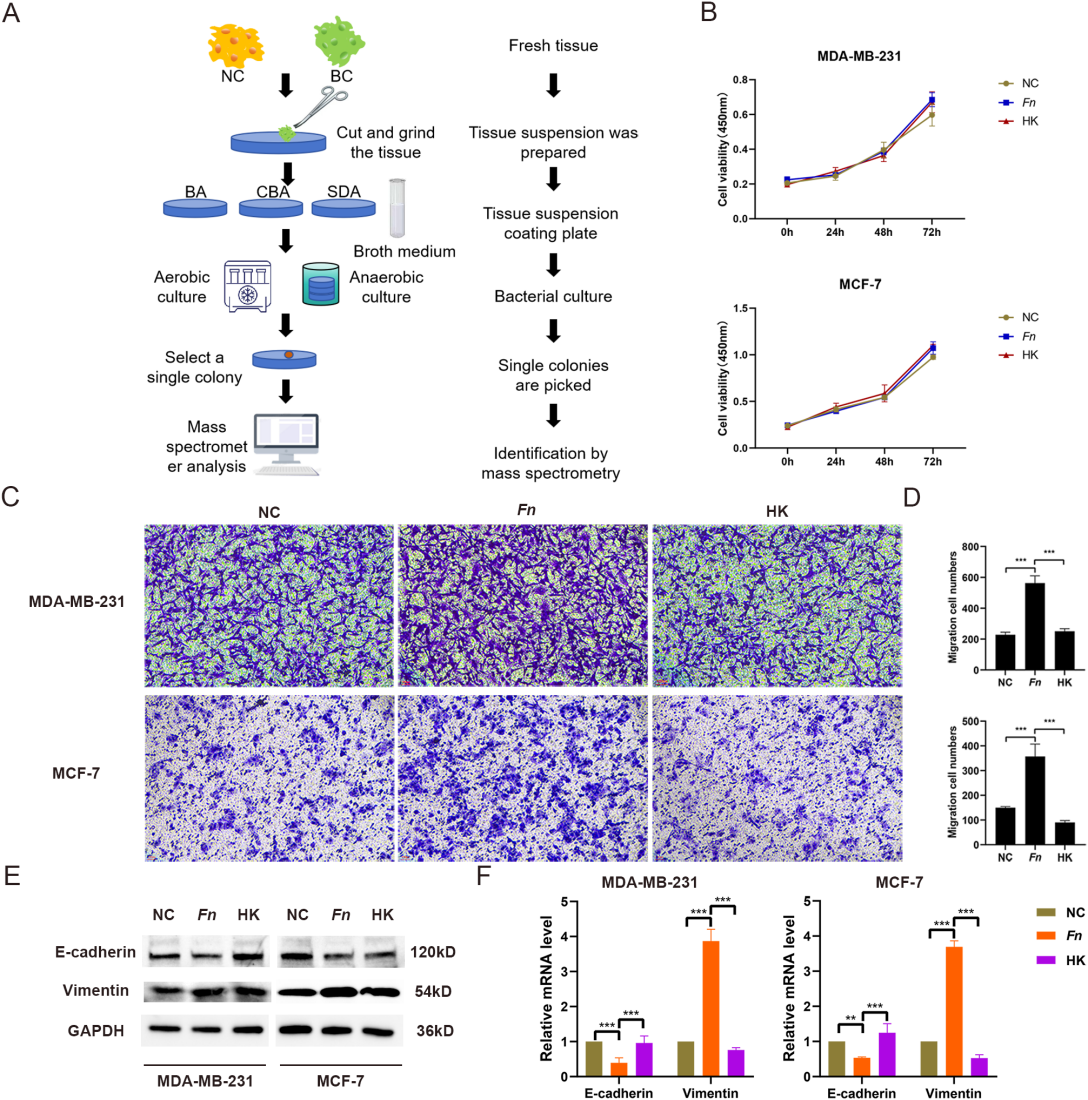
Isolation, culture, and functional identification of F. nucleatum. (A) Flowchart illustrating the bacterial isolation and culture process from tumor tissues. NC represents adjacent healthy tissue; BC indicates breast cancer tissue. (B) Results of the CCK-8 assay evaluating the effect of F. nucleatum infection on BC cell proliferation. MDA-MB-231 and MCF-7 cells were seeded in 96-well plates and infected with F. nucleatum (MOI = 50:1) for 0, 24, 48, and 72 hours. Fresh culture medium was replaced, and CCK-8 reagent was added. The plates were incubated at 37°C for 1 hour, and absorbance was measured at a wavelength of 450 nm. NC refers to the untreated group; Fn represents the F. nucleatum-infected tumor cell group (MOI = 50:1); HK denotes the heat-inactivated F. nucleatum group. (C, D) Representative images (C) and statistical analysis (D) of the Transwell assay assessing the effect of F. nucleatum infection on BC cell migration. (E, F) WB and RT-qPCR analysis of the impact of F. nucleatum infection on the expression levels of EMT-related proteins (E) and mRNA (F) in BC cells. MDA-MB-231 and MCF-7 cells were seeded in 6-well plates and infected with either F. nucleatum or heat-inactivated F. nucleatum (MOI = 50:1) for 48 hours, followed by cell collection for Transwell assays and WB analysis. **P < 0.01, ***P < 0.001.

To determine the presence of *F. nucleatum* in BC tissues, bacterial isolation and culture were conducted using fresh BC tissues and healthy breast tissues (**Figure 1**). Colonies were identified through mass spectrometry, which confirmed the presence of *F. nucleatum* in BC tissues. Subsequently, an *in vitro* model was established using *F. nucleatum* isolated from clinical samples to infect MDA-MB-231 and MCF-7 cells at a multiplicity of infection of 50:1. The impact of *F. nucleatum* on BC cell proliferation and migration was assessed through CCK-8, Transwell, and scratch assays. As anticipated, the results indicated that while *F. nucleatum* infection did not affect BC cell proliferation compared to the control and heat-killed (HK) bacteria groups (**Figure 1**), it significantly enhanced the migratory capacity of breast cancer cells (*p* < 0.01) (**Figures 1**, **Supplementary Figure 1**). Furthermore, the expression levels of the epithelial marker E-cadherin and the mesenchymal marker Vimentin in MDA-MB-231 and MCF-7 infection models were analyzed through Western blot analysis and RT-qPCR. The findings unveiled significant downregulation of E-cadherin and upregulation of Vimentin in the *F. nucleatum* infection group compared to the negative control (NC) group, while inactivation of *F. nucleatum* reversed these gene expression changes (**Figures 1**).

### *F. nucleatum* promotes BC cell migration via miR-21-3p

**Figure 2:**
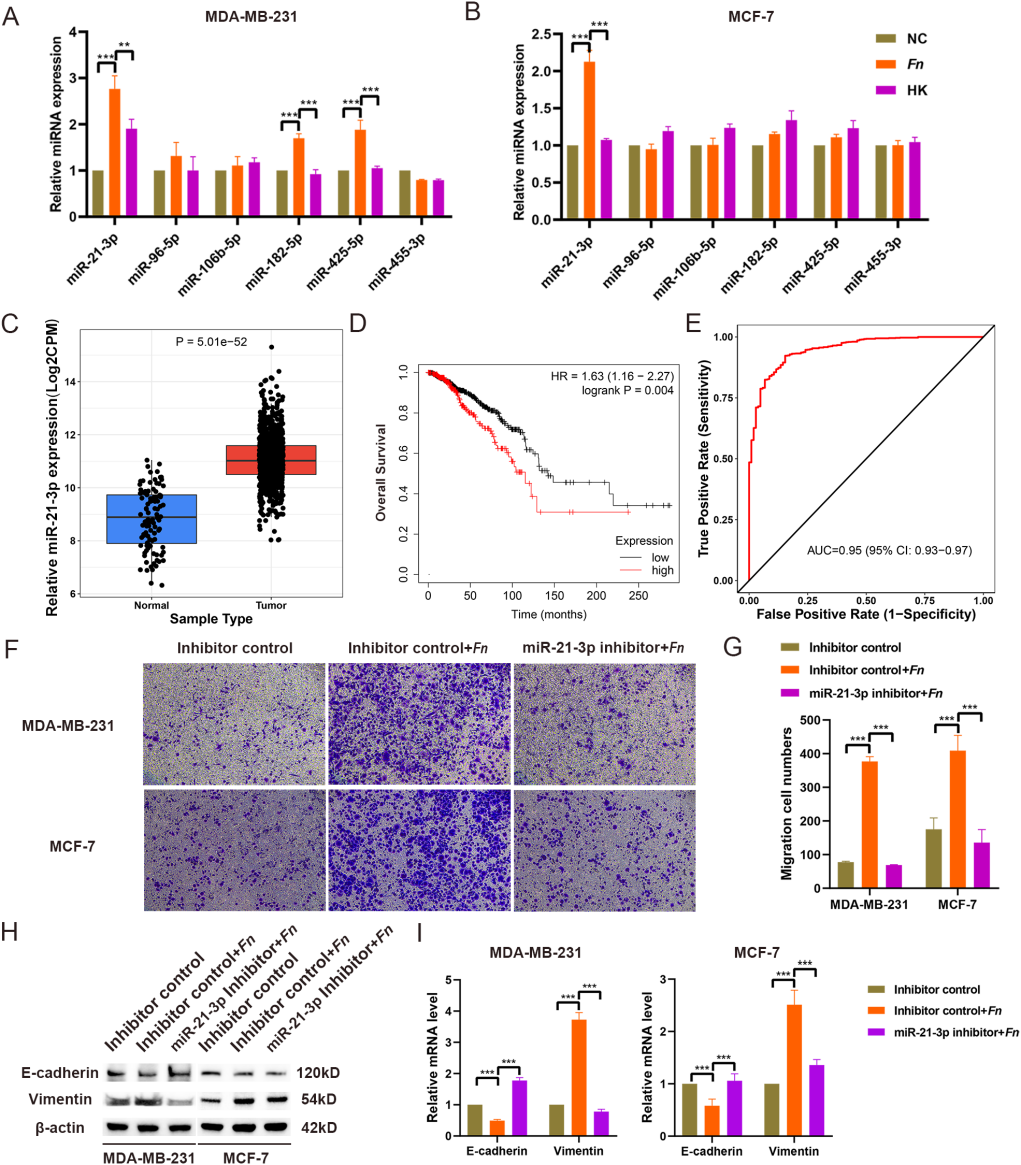
Selection and identification of miR-21-3p. (A, B) The expression levels of miRNAs in MDA-MB-231 (A) and MCF-7 (B) cell models infected with F. nucleatum were assessed using RT-qPCR. (C) The expression of miR-21-3p in BC tissues was evaluated using the CancerMIRNome database. (D) The effect of miR-21-3p expression on the overall survival of BC patients was analyzed using the KM Plotter database. (E) The role of miR-21-3p in BC diagnosis was determined through the CancerMIRNome database. (F, G) Representative images (F) and statistical analyses (G) illustrating the impact of miR-21-3p knockdown on the migratory ability of F. nucleatum-infected BC cells. (H, I) The influence of miR-21-3p knockdown on the expression of EMT-related proteins (H) and mRNA (I) in F. nucleatum-infected BC cells was evaluated. MDA-MB-231 and MCF-7 cells were seeded in 6-well plates and subjected to siRNA transfection. Following 6–8 hours of transfection, the medium was replaced, and the cells were infected with F. nucleatum (MOI = 50:1) for 48 hours. Cells were subsequently collected for Transwell assays and WB analysis. The group transfected with inhibitor control siRNA is referred to as “Inhibitor control.” The group that underwent F. nucleatum infection after transfection with the inhibitor control plasmid is labeled as “Inhibitor control + Fn.” The group transfected with the miR-21-3p knockdown plasmid followed by F. nucleatum infection is designated as “miR-21-3p inhibitor + Fn.”. **P < 0.01, ***P < 0.001.

In previous studies conducted by our research group, miRNA sequencing was performed on tissue samples collected from five BC patients and five healthy breast tissues (31). An analysis of the sequencing data and existing literature initially yielded six miRNAs (**Supplementary Table 3**). RT-qPCR validation demonstrated that in MDA-MB-231 cells, miR-21-3p, miR-182-5p, and miR-425-5p levels were significantly higher in the *F. nucleatum* infection group compared to NC and HK groups. In MCF-7 cells, only the expression level of miR-21-3p was increased (**Figures 2**). As a result, miR-21-3p was selected for further experiments.

Initially, online database analysis was used to evaluate the expression of miR-21-3p in BC tissues and its impact on overall survival in BC patients. CancerMIRNome database analysis uncovered that the expression level of miR-21-3p was significantly higher in BC tissues compared to healthy tissues (*p* < 0.05) (**Figure 2**). At the same time, Kaplan-Meier analysis illustrated that high miR-21-3p expression was significantly associated with shorter survival in BC patients (**Figure 2**). Moreover, ROC analysis indicated an AUC value of 0.95 for miR-21-3p, suggesting its high diagnostic value in BC (**Figure 2**). These results collectively highlight the critical role of miR-21-3p in BC development.

To explore the contribution of miR-21-3p in enhancing *F. nucleatum*-mediated BC cell metastasis, miR-21-3p knockdown BC cell lines were established using siRNAs. Transwell and scratch assays were utilized to investigate the effect of miR-21-3p knockdown on *F. nucleatum*-induced BC cell migration. Interestingly, the results demonstrated that cells transfected with the miR-21-3p inhibitor exhibited significantly reduced migratory ability compared to those transfected with the inhibitor control in the *F. nucleatum* infection group (**Figures 2**). Similarly, scratch assay results indicated a decreased migratory rate in cells transfected with the miR-21-3p inhibitor compared to the inhibitor control group in the *F. nucleatum* infection group (**Supplementary Figure 2**). Taken together, these results suggest that miR-21-3p inhibition attenuates *F. nucleatum*-induced BC cell migration. Additionally, Western blot analysis and RT-qPCR demonstrated that miR-21-3p suppression significantly impeded *F. nucleatum*-induced EMT in breast cancer cells (**Figures 2**).

### FOXO3 is a downstream target of miR-21-3p

**Figure 3:**
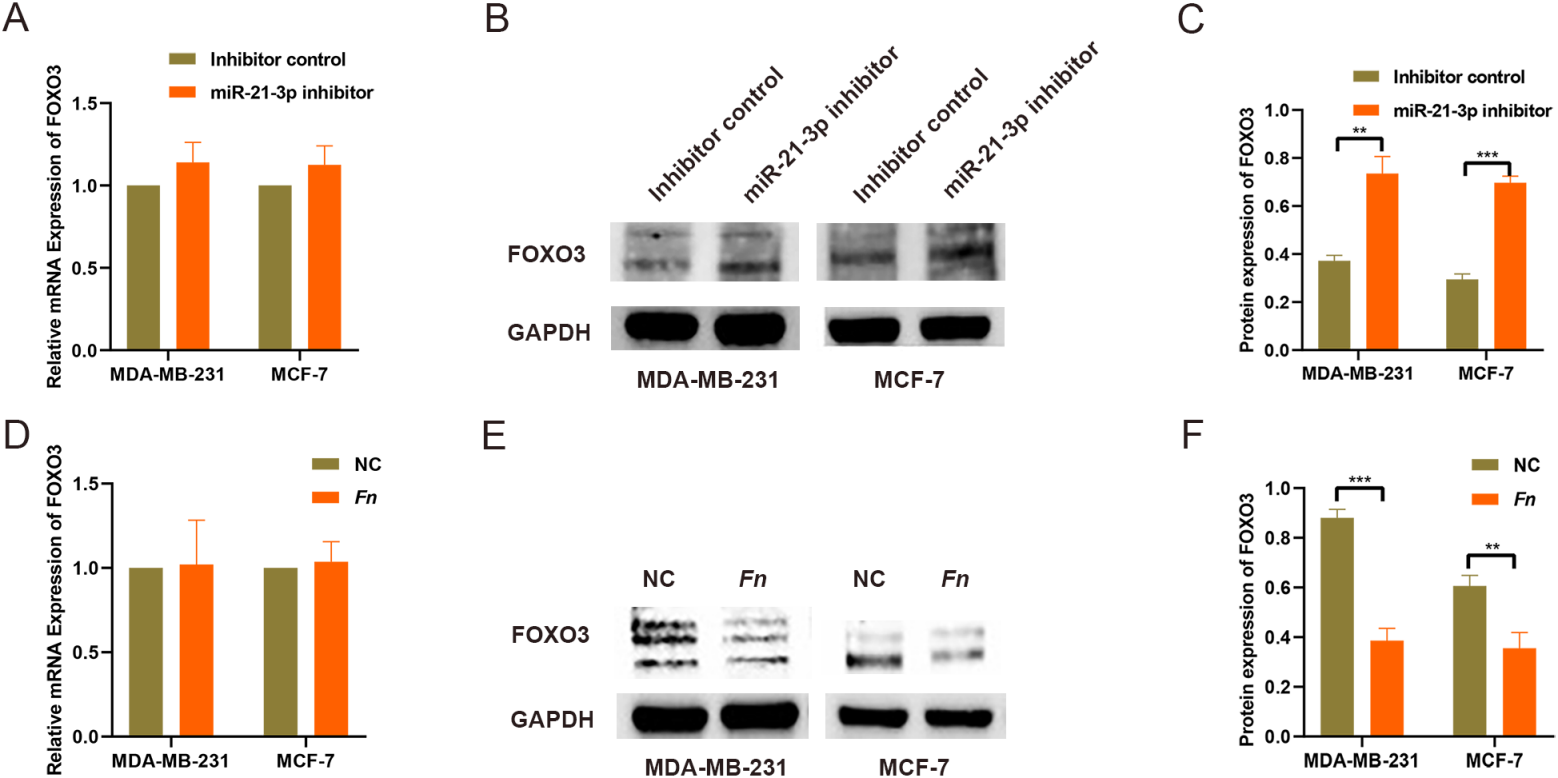
Screening and validation of downstream target genes regulated by miR-21-3p. (A) The impact of miR-21-3p knockdown on FOXO3 mRNA expression was analyzed using RT-qPCR. (B, C) WB was employed to assess FOXO3 protein expression levels in BC cells following miR-21-3p knockdown, with representative images shown in (B) and statistical data in panel (C, D). The effect of F. nucleatum infection on FOXO3 mRNA expression was evaluated using RT-qPCR. (E, F) WB was utilized to determine the effect of F. nucleatum infection on FOXO3 protein expression in BC cells, with results presented in (E) and statistical analysis in (F). **P < 0.01, ***P < 0.001.

To explore potential downstream targets of miR-21-3p, predictions were performed using the miRDB online database, which identified FOXO3 as a target gene, whilst the presence of binding sites was corroborated through TargetScan database analysis (**Supplementary Figure 3**). Of note, previous studies have established the tumor-suppressive role of FOXO3 in various cancers. In this study, miR-21-3p expression was markedly upregulated in both BC tissues and *F. nucleatum*-infected BC cell models. Consequently, FOXO3 was hypothesized to be a downstream target of miR-21-3p. Validation through RT-qPCR and Western blot analysis revealed no significant change in FOXO3 mRNA levels. However, the protein expression of FOXO3 was significantly higher in the miR-21-3p knockdown groups compared to controls (**Figures 3**). Furthermore, *F. nucleatum* infection lowered FOXO3 protein levels in BC cells without affecting mRNA expression (**Figures 3**). Taken together, these findings signal that FOXO3 functions as a downstream target of miR-21-3p.

### FOXO3 mediates the pro-migratory effects of miR-21-3p

**Figure 4:**
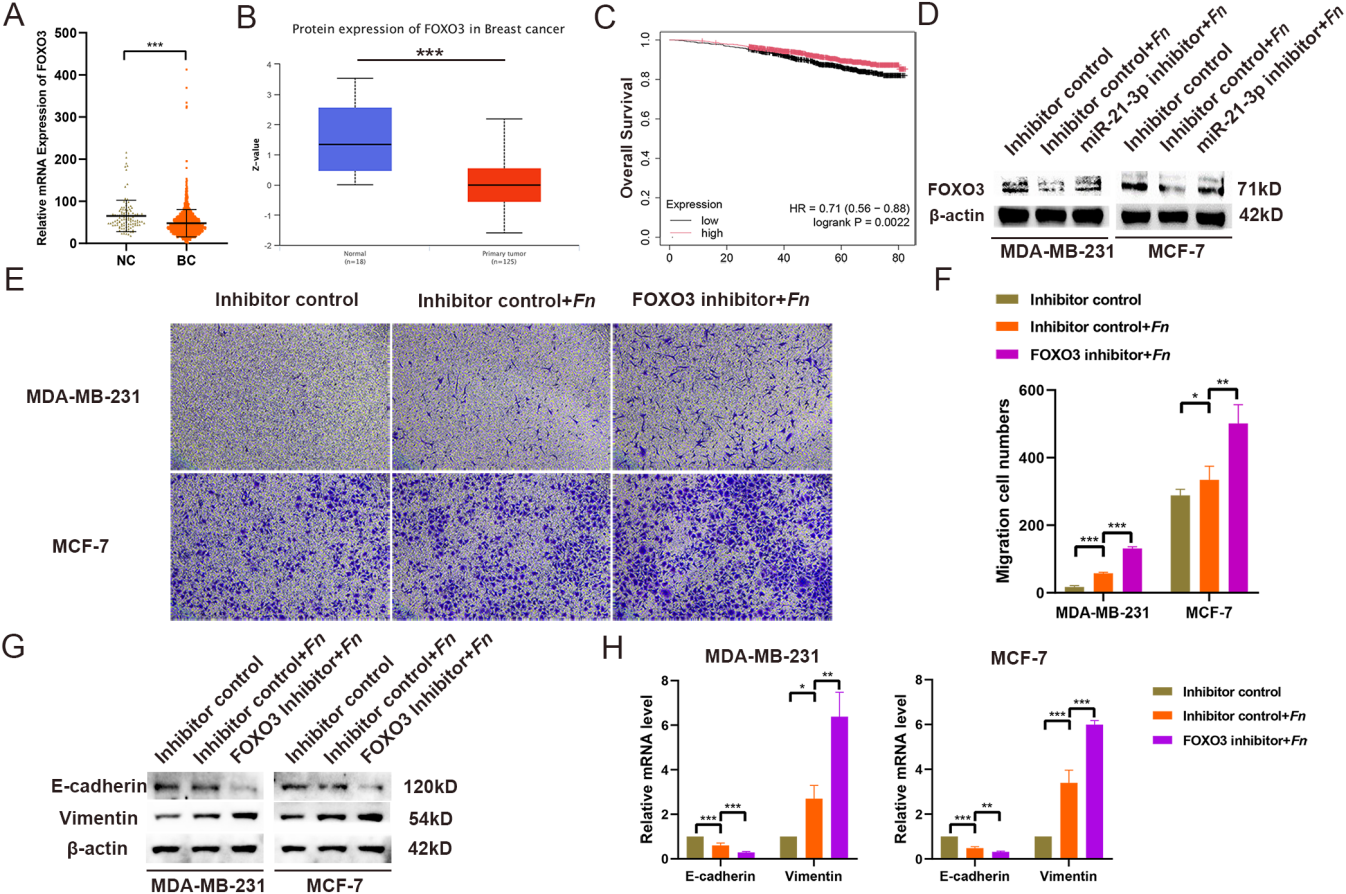
FOXO3 Expression and Functional Characterization. **(A)** The expression of FOXO3 was analyzed using data from the TCGA BRCA database. **(B)** Protein expression levels of FOXO3 were evaluated through the UALCAN database. **(C)** The effect of FOXO3 expression on the overall survival of BC patients was assessed using the KM Plotter database. **(D)** Western blotting (WB) was employed to investigate the effects of *F*. *nucleatum* infection and miR-21-3p knockdown on FOXO3 protein expression in BC cells. **(E, F)** Representative images **(E)** and statistical analyses **(F)** from Transwell assays of BC cells infected with *F*. *nucleatum* and subjected to miR-21-3p knockdown. **(G, H)** The impact of FOXO3 knockdown on the expression of EMT-related proteins **(G)** and mRNA **(H)** in *F*. *nucleatum*-infected BC cells was evaluated. *P < 0.05, **P < 0.01, ***P < 0.001.

To validate the involvement of FOXO3 as a miR-21-3p downstream target in *F. nucleatum*-induced BC cell migration, further experiments were conducted. TCGA database analysis indicated significantly lower FOXO3 expression levels in breast cancer tissues compared to adjacent non-cancerous tissues (**Figure 4**). Consistently, the results of UALCAN database analysis corroborated reduced FOXO3 protein levels in breast cancer tissues (**Figure 4**). More importantly, low FOXO3 levels were significantly associated with poorer overall survival in BC patients (**Figure 4**). Subsequent experiments assessing the impact of miR-21-3p knockdown on FOXO3 protein expression in *F. nucleatum*-infected BC cells revealed that miR-21-3p inhibition attenuated the suppressive effect of *F. nucleatum* on FOXO3 (**Figure 4**), implying that miR-21-3p may promote BC cell migration through FOXO3 inhibition. To validate these observations, a FOXO3 knockdown cell model was generated. Transwell assays demonstrated that FOXO3 knockdown significantly promoted BC cell migration (**Figures 4**), in line with the results of the scratch assays (**Supplementary Figure 4**). Likewise, the results of Western blot analysis and RT-qPCR delineated that FOXO3 knockdown further elevated EMT-related gene expression at both the protein and mRNA levels (**Figure 4**).

## Discussion

As is well documented, breast cancer (BC) is the most prevalent malignant tumor among women worldwide, with the number of new cases increasing annually. In China, its incidence continues to rise, making it a major cause of cancer-related deaths among women (1, 2). The development of distant metastasis significantly complicates the treatment of BC and contributes to its high mortality rate. Therefore, there is a pressing need to identify new risk factors for metastasis and elucidate their underlying mechanisms for improving therapeutic strategies for metastatic BC.

*F. nucleatum* has been widely implicated in colorectal cancer, oral cancer, and BC tissues, playing a vital role in their progression. Herein, *F. nucleatum* was isolated and cultured from tumor and adjacent non-tumor samples from BC patients. Functional studies indicated that while isolated *F. nucleatum* infection did not affect the proliferation of BC cells, it significantly promoted their migration. This effect was negated by the heat inactivation of *F. nucleatum*, suggesting that bacterial viability is a critical factor influencing tumor cell migration and that eliminating intratumoral bacteria might serve as an adjunctive strategy in cancer therapy.

Tumor metastasis represents a significant cause of mortality in patients with BC, wherein miRNAs play a pivotal mediating role. Prior investigations by our research team employed sequencing methods to identify miRNAs that exhibit differential expression between BC tissues and healthy breast tissues (31), demonstrating that the expression levels of miR-21-3p, miR-96-5p, and miR-182-5p were significantly elevated in the BC tissues. To validate these sequencing results and explore the mechanism by which *F. nucleatum* promotes metastasis, *F. nucleatum*-infected MDA-MB-231 and MCF-7 cell *in vitro* models were established for RT-qPCR validation. The results unraveled that miR-21-3p expression was significantly upregulated in both models. Noteworthily, miR-21 is a potential diagnostic, prognostic, and predictive biomarker for various cancer types, including BC (32). However, previous research has primarily focused on miR-21-5p, with limited studies examining the role of miR-21-3p in BC, which have noted correlations between elevated miR-21-3p expression levels, BC progression, and shorter overall survival (33). consistent with the results of the bioinformatics analysis. Moreover, a miR-21-3p inhibitor was used to construct miR-21-3p knockdown cell models to explore the role of miR-21-3p in *F. nucleatum*-induced migration of MDA-MB-231 and MCF-7 cells. Transfection with the miR-21-3p inhibitor significantly down-regulated miR-21-3p expression and inhibited cell migration, suggesting that downregulation of miR-21-3p can mitigate *F. nucleatum*-induced migration of MDA-MB-231 and MCF-7 cells and that miR-21-3p mediates the pro-migratory effect of *F. nucleatum* on breast cancer cells. The current study also examined the impact of *F. nucleatum* infection and miR-21-3p knockdown on EMT in breast cancer cells. The results showed that *F. nucleatum* infection significantly inhibited the expression of the epithelial marker E-cadherin, while promoting Vimentin expression, thus facilitating EMT in breast cancer cells. However, this process was inhibited following the down-regulation of miR-21-3p expression, suggesting that miR-21-3p mediates the pro-migratory effect of *F. nucleatum* on breast cancer cells through the promotion of tumor cell EMT.

Previous studies have reported the downregulation of FOXO3 in various cancers, where it acts as a tumor suppressor (34). In addition, FOXO3 has been linked to tumor metastasis, given its inactivation induces Snail expression and enhances tumor cell EMT, thereby facilitating invasion and metastasis, as observed in renal clear cell carcinoma (35). To verify whether FOXO3 is a target gene of miR-21-3p, TargetScan online database predictions were utilized to assess sequence matching, revealing a binding site in the 3’ UTR of FOXO3. Further studies demonstrated an upregulation of miR-21-3p and concurrent downregulation of FOXO3 in *F. nucleatum*-infected MDA-MB-231 and MCF-7 cells, with a negative correlation noted between their expression levels. This indicates that FOXO3 is a downstream target of miR-21-3p. Given that *F. nucleatum* infection promotes the migration of MDA-MB-231 and MCF-7 cells and that miR-21-3p mediates this process, the role of FOXO3 as a downstream target gene was explored. Liposome transfection was used to introduce FOXO3 siRNA into MDA-MB-231 and MCF-7 cells to inhibit FOXO3 expression. The results showed that FOXO3 siRNA increased cell migration in *F. nucleatum*-infected cells, further promoting the expression of EMT-related genes. This finding suggests that inhibiting FOXO3 expression in MDA-MB-231 and MCF-7 cells reverses the protective effect of miR-21-3p downregulation against *F. nucleatum*-induced EMT and cell migration. In summary, inhibition of FOXO3 expression enhances *F. nucleatum*-induced cell migration.

Overall, this study demonstrated that while *F. nucleatum* infection does not promote cell proliferation in MDA-MB-231 and MCF-7 cells, it enhances BC cell migration. The promotion of BC cell migration by *F. nucleatum* infection can be attributed to the enhancement of breast cancer cell EMT through the miR-21-3p/FOXO3 axis. Furthermore, the use of inactive *F. nucleatum* can reverse this process. Collectively, this research indicates that *F. nucleatum* enhances cell EMT via the miR-21-3p/FOXO3 signaling axis, thereby promoting BC cell migration. Finally, the results suggest that eradicating *F. nucleatum* might serve as a promising therapeutic strategy for breast cancer.

## Data Availability

The data presented in the study are deposited in the GEO repository, accession number GSE298584.

## Supplementary material

Supplementary Figure 1 The effect of *F. nucleatum* infection on BC cell migration was assessed using a wound healing assay.

Supplementary Figure 2 The influence of miR-21-3p knockdown on the promotion of BC cell migration by *F. nucleatum* infection was evaluated through a wound healing assay.

Supplementary Figure 3 The TargetScan database was utilized to analyze the base pairing between miR-21-3p and FOXO3.

Supplementary Figure 4 The role of FOXO3 knockdown in modulating the promotion of BC cell migration by *F. nucleatum* infection was investigated using a wound healing assay.
