# Supplementary figures and images for: Fusobacterium nucleatum promotes metastasis of breast cancer via the miR-21-3p/FOXO3 axis
Source: Front Oncol. 2025 Jun 16;15:1530269. doi: 10.3389/fonc.2025.1530269 (PMC12206713; doi:10.3389/fonc.2025.1530269)

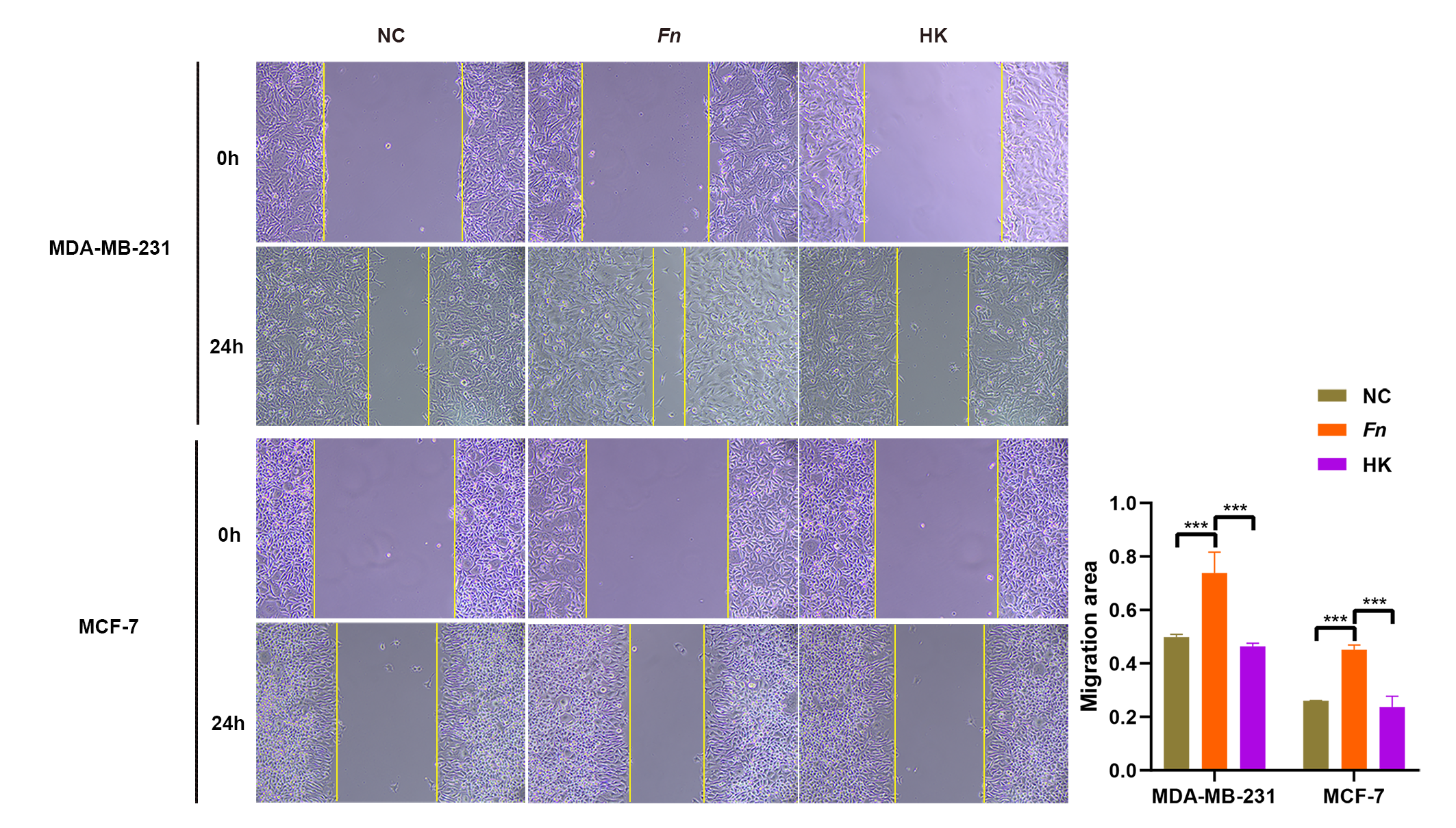

Supplement: Supplementary Figure 1 — The effect of F. nucleatum infection on BC cell migration was assessed using a wound healing assay. [file Image1.tif]

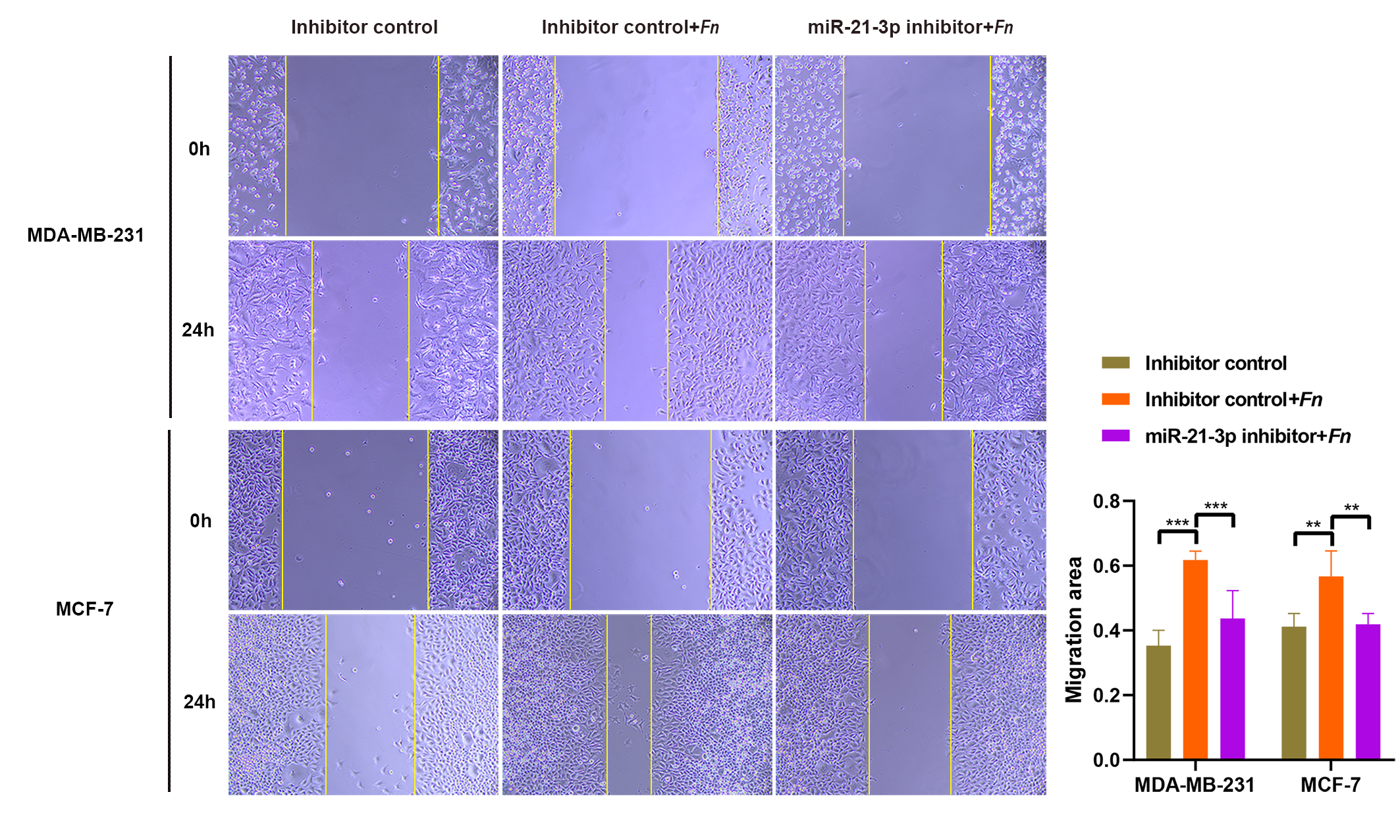

Supplement: Supplementary Figure 2 — The influence of miR-21-3p knockdown on the promotion of BC cell migration by F. nucleatum infection was evaluated through a wound healing assay. [file Image2.tif]

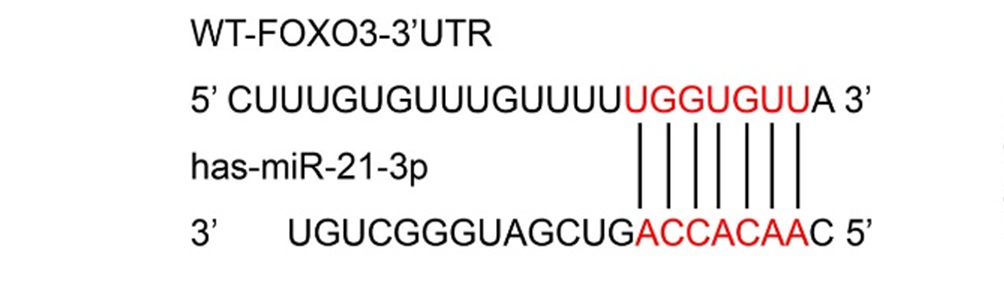

Supplement: Supplementary Figure 3 — The TargetScan database was utilized to analyze the base pairing between miR-21-3p and FOXO3. [file Image3.tif]

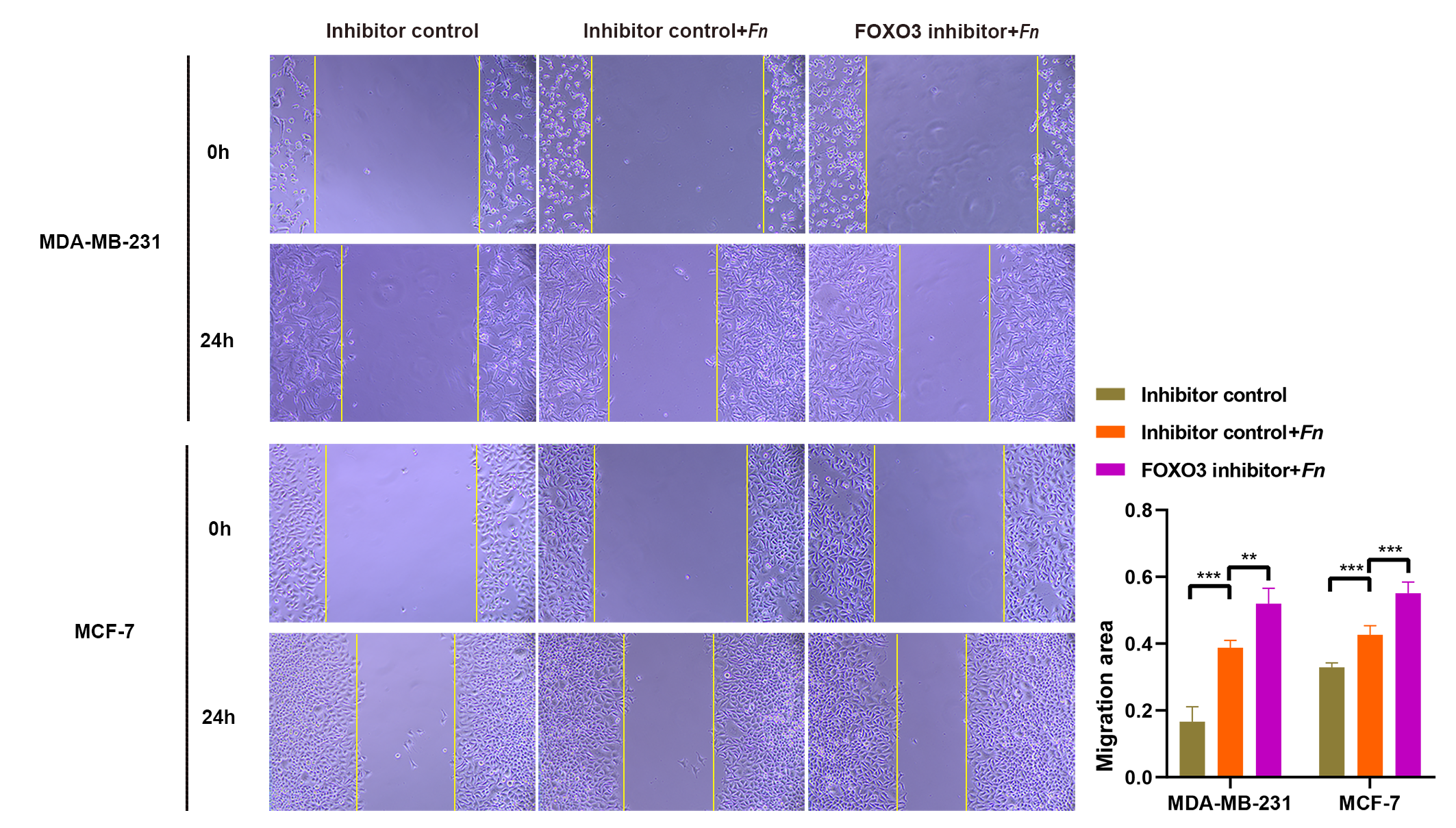

Supplement: Supplementary Figure 4 — The role of FOXO3 knockdown in modulating the promotion of BC cell migration by F. nucleatum infection was investigated using a wound healing assay. [file Image4.tif]
